# Gender differences in factors associated with symptoms of depression among high school students: an examination of the direct and indirect effects of insomnia symptoms and physical activity

**DOI:** 10.1080/21642850.2019.1615926

**Published:** 2019-05-15

**Authors:** Eva Langvik, Ingvild Saksvik-Lehouillier, Leif Edward Ottesen Kennair, Torhild Anita Sørengaard, Mons Bendixen

**Affiliations:** Department of Psychology, Norwegian University of Science and Technology, Trondheim, Norway

**Keywords:** Mental health and disorder < AREAS OF EXPERTISE, sleep disorder < AREAS OF EXPERTISE, children and adolescents < POPULATION OF EXPERTISE

## Abstract

**Objective:**

Scant research exists on the gender-specific association between physical activity, insomnia symptoms and depressive symptoms among adolescents. The present study investigates the direct and indirect association of insomnia and physical activity with symptoms of depression.

**Design:**

In a community-based sample (*N* = 1485) we investigated factors associated with symptoms of depression focusing on insomnia. The study also included measures of physical activity and controlled for parental work- and sexual minority status. Body mass Index (BMI) was calculated for a sub-sample (*n* = 617) reporting weight and height.

**Results:**

The results showed that self-reported insomnia was highly prevalent, and the association between insomnia and depression was strong. The association between insomnia and depression was significantly stronger for girls than for boys. The effect of physical activity was substantially weaker compared to insomnia. Insomnia mediated the relationship between physical activity and depression for both boys and girls. Despite expectation based on the existing literature, BMI showed no association with symptoms of depression or physical activity.

**Conclusion:**

The results address the importance of a gender-specific approach when investigating mental health among adolescents. Given the high prevalence, interventions aimed at reducing insomnia is important in the prevention of mental illness, especially among girls.

Depression among adolescents constitutes a major health challenge and means should be taken to minimize the cost both on the individual and societal level (Carrellas, Biederman, & Uchida, 2017; Merikangas, Nakamura, & Kessler, 2009). Worldwide, one third of all youth experience a mental disorder across their lifetime (Merikangas et al., 2009), and in Norway today, 15–20 percent of children between 3 and 18 years have reduced function due to symptoms of mental disorders such as anxiety and depression (Norwegian Institute of Public Health, 2016). The frequency of mental illness amongst adolescents and potential long-term consequences warrants the investigation of both risk factors and protective factors. Depression is highly prevalent among adolescents, and even sub-threshold depression is associated with functional impairment and suicidality, warranting early interventions aimed at mitigating these adverse outcomes (Carrellas et al., 2017). In children, physical activity predicts fewer symptoms of depression (Zahl, Steinsbekk, & Wichstrøm, 2017), while research on the relationship between physical activity and depressive symptoms in adolescents has produced mixed results (e.g. Arat & Wong, 2017) and few studies have included measures of insomnia. Despite the substantial research on the relationship between physical activity and depression (e.g. Arat & Wong, 2017; Mammen & Faulkner, 2013; Zahl et al., 2017), and on depression and insomnia (Alvaro, Roberts, & Harris, 2014; de Zambotti, Goldstone, Colrain, & Baker, 2018; Roberts & Duong, 2013), studies on gender-specific effects of physical activity and insomnia on level of depression among adolescents are scarce.

Adolescence often leads to changes in sleep patterns; including sleep problems, especially late bed times, and short sleep duration (Hysing, Pallesen, Stormark, Lundervold, & Sivertsen, 2013). Considerable research has considered the natural delay in the sleep/wake cycle that normally occurs during adolescence across countries and cultures (Gradisar, Gardner, & Dohnt, 2011). The prevalence of delayed sleep phase syndrome is particularly high and is associated with elevated anxiety and depression symptoms along with unhealthy lifestyle (Saxvig, Pallesen, Wilhelmsen-Langeland, Molde, & Bjorvatn, 2012). The prevalence of insomnia is high among adolescents (Hysing et al., 2013), and has in general increased during the past decade (Pallesen, Sivertsen, Nordhus, & Bjorvatn, 2014). Criteria for insomnia according to the Diagnostic and Statistical Manual of Mental Disorders, Fourth Edition (DSM-IV) (AmericanPsychiatricAssociation, 2000) includes difficulty initiating or maintaining sleep or non-restoratory sleep for at least one month, and/or causing clinically significant distress in social, education or other important areas of functioning. Insomnia is thus a problem that affects both nighttime sleep and daytime functioning. Insomnia disorder is particularly common among older adolescents. However, despite having a prevalence comparable to depression, insomnia has received limited attention compared to other mental health problems (de Zambotti et al., 2018). Insomnia represents an important predictor of school absenteeism among adolescents, even when controlling for other psychological factors (Bauducco, Tillfors, Özdemir, Flink, & Linton, 2015).

Evidence suggests that physical activity can prevent  the development of depression (Mammen & Faulkner, 2013), and that higher levels of physical activity among children and young adolescents are associated with fewer symptoms of depression (Kremer et al., 2014). Regular physical activity may also reduce the risk for future increase in depressive symptoms, and may have a protective effect against the onset of depression (Jerstad, Boutelle, Ness, & Stice, 2010). Physical activity is also linked to overweight, a factor found to independently predict future symptoms of depression (Cho et al., 2018). Previous research shows that both exercise and sleep play significant roles in the development and maintenance of depression (Lopresti, Hood, & Drummond, 2013). Among adolescent athletes, researchers found that exercise was related to better sleep and fewer symptoms of insomnia (Brand et al., 2010). There is a robust positive relationship between physical exercise, and sleep (Kredlow, Capozzoli, Hearon, Calkins, & Otto, 2015; Lang et al., 2016), and exercise is associated with better sleep across different age groups (Youngstedt & Kline, 2006). A recent large-scale study showed that the decrease in physical activity and increase in depressive symptoms is most pronounced around the age of 15–16 and that gender differences are prevalent both in levels of depression and physical activity (Baldursdottir, Valdimarsdottir, Krettek, Gylfason, & Sigfusdottir, 2017). Reviews of the literature on the effectiveness of physical activity interventions suggest that increased activity promotes better mental health (Camero, Hobbs, Stringer, Branscum, & Taylor, 2012) and may prevent depression (Zahl et al., 2017). A recent study on adolescents observed a negative association between physical activity and depression while the association between activity and anxiety was positive (He, Paksarian, & Merikangas, 2018). However, the results regarding physical activity and depression among adolescents remain unclear, as most of the studies do not consider gender differences nor have they controlled for insomnia. The reciprocal causal relationship between symptoms of depression and symptoms of insomnia among adolescents has received little attention so far, and prospective studies on this subject have shown mixed results. In one study, a reciprocal relationship between insomnia and depression was observed (Roberts & Duong, 2013)*.* In another study, depression predicted sleep problems, but sleep problems as a predictor of depression were not supported (Hayley et al., 2015). The primary causal role of insomnia in the development of depression is further supported by longitudinal studies showing that insomnia predicts symptoms of depression, while symptoms of depression have failed to predict future insomnia (Johnson, Roth, & Breslau, 2006). Although insomnia is related to depression (Alvaro et al., 2014; Roth, 2007), insomnia is also an independent concept related to suicidality and substance abuse (de Zambotti et al., 2018) and absenteeism among adolescents (Bauducco et al., 2015). As sleep problems in general both predict and are predicted by depression, screening children for sleep problems has been suggested as an effective avenue for reducing the burden of mental illness among adolescents (Shanahan, Copeland, Angold, Bondy, & Costello, 2014). Randomized-controlled trials have showed that internet-delivered CBT for insomnia also reduced symptoms of anxiety and depression (Hagatun et al., 2018). Given the close and intertwined relationship between insomnia and depression, early detection and treatment of insomnia might limit the risk of developing depression among children and adolescents (Baglioni et al., 2011).

## Gender differences in depression, insomnia and physical activity

The lifetime prevalence of affective disorders among women is almost doubled compared to men (Faravelli, et al., 2013), and for major depression, the sex difference in prevalence ratio is even stronger (Delisle, Beck, Dobson, Dozois, & Thombs, 2012). The difference in rates of depression emerge during mid-puberty and persists throughout adulthood (Piccinelli & Wilkinson, 2000; Whalen et al., 2016), and adolescence is suggested to be the key developmental period for the emergence of gender differences in depression (Sequeira, Lewis, Bonilla, Smith, & Joinson, 2017). Social factors, like weight-related concerns, have been suggested as possible explanations of the emergence of gender-differences in depression during adolescence (Vaughan & Halpern, 2010), while others focus on physiological explanations (Parker & Brotchie, 2010) and the role of sex-hormones (Barth, Villringer, & Sacher, 2015). Considerable cross-national differences exist and compared to other European countries the level of depressive symptoms is lowest in Norway (Van de Velde, Bracke, & Levecque, 2010). Still, adolescent girls report moderately more symptoms of depression than boys in Norway (Bendixen, Daveronis, & Kennair, 2018).

The relationship between physical activity and depression is complex, and boys and girls show differential developmental trajectories (Ames & Wintre, 2016). Further, physical activity is inevitably linked to obesity, which has a gender-specific social association with depression (Needham & Crosnoe, 2005). Among adolescents, gender differences are observed not only in depression (Altemus, Sarvaiya, & Neill Epperson, 2014) and insomnia (de Zambotti et al., 2018), but also in vulnerability to sleep deprivation (Short & Louca, 2015), psychological impact of obesity (Mulugeta, Zhou, Power, & Hyppönen, 2018) and physical activity (Baldursdottir et al., 2017). This study adds to the existing literature by investigating the gender-specific association between insomnia, physical activity and obesity in a large community sample, controlling for factors previously linked to depression in this population, i.e. sexual minority status and family background (Bendixen et al., 2018).

## Present study and hypothesis

The current study aims to investigate the gender-specific associations between insomnia, physical activity, and depression in a large, community-based sample. We hypothesize that insomnia will be strongly associated with symptoms of depression, and that higher levels of physical activity will be associated with less symptoms. Further, we expect BMI to be associated with both physical activity and depression. Finally, we want to examine whether the associations between physical activity, insomnia and symptoms of depression are gender specific. Finally, we want to examine to what extent the relationship between physical activity and depression is mediated by insomnia, and to explore the possible mediating effect of physical activity on the relationship between insomnia and depression.

## Method

### Study design and participants

The study follows a cross-sectional design and was carried out among high school students in Central Norway during May/June 2013, November/December 2013 and May/June 2014. The data was screened for inconsistent, unlikely, monotonous and extreme responding, reflecting lack of motivation. The final sample consisted of 1485 students (58.7% girls and 41.3% boys), aged between 16 and 21 (Mean age was 17.7 for both girls and boys). More girls (12.5%) than boys (8.4%) reported non-heterosexual orientation (sexual minority status) and almost one-in-five students reported that at least one parent was not currently employed. The sample is considered representative of high-school students in Norway. We did not record the number of students invited to participate. Hence, the exact response rate is not known for this study, but a in a comparable study of sexual harassment using identical design and methodology (see Kennair & Bendixen, 2012), the response rate for this study is estimated to be 50% or higher (see also Bendixen et al., 2018 for a comparison of study characteristics).

### Procedure

Students, their parents and the school staff received written information about the study, stating the purpose and content of the project. The school administered the written information and informed consent form, and students received a login code in exchange for returning the consent form to get access to the computer-assisted survey. Students could respond to the questionnaire on their designated computer at home or in the classroom. Arrangements for group administration at school ensured anonymity and confidentiality. Throughout the weeks that the survey took place the schools’ public health nurses were available for consultation. The Regional Committee for Medical and Health Research Ethics approved the procedure.

### Materials

#### Symptoms of insomnia

The Bergen Insomnia Scale (BIS) (Pallesen et al., 2008) contains six items that assess symptoms of insomnia based on the American Psychiatric Association’s (1994; APA) Diagnostic and Statistical Manual of Mental Disorders-IV. The participants indicated how many days a week (0–7) they have struggled with six specific symptoms of insomnia during the last month. The first three items measure sleep onset, sleep maintenance, and early morning wakening insomnia, respectively. The last three items refer to not feeling adequately rested, experiencing daytime impairment due to poor sleep and being dissatisfied with current sleep. In the main analyses we used continuous scores of insomnia problems, however, we also calculated prevalence of insomnia among boys and girls, where the diagnostic criteria of insomnia are met with a score of > 2 on one or more of the first four items (nocturnal symptoms) and a score of > 2 on one or more of the last two items (daytime symptoms). The scale has good convergent and discriminative validity, and satisfying psychometric properties (Pallesen et al., 2008). Internal consistency for the 6-item scale was good (*α *= .79).

#### Physical activity (Exercise)

Two questions measured the level of physical activity. First, the students rated their partaking in the school’s mandatory physical education classes on a four-item scale 1 (*never*), 2 (*sometimes*), 3 (*most of the time*), and 4 (*always*). They then rated their frequency of exercise outside of school’s physical education on a five-item scale 1 (*never*), 2 (*once a week*), 3 (*between 2 and 3 times a week*), 4 (*between 4 and 5 times a week*), and 5 (*between 6 and 7 times a week*). The two indicators of physical activity correlated moderately (*r* = .25). The scores were summed and averaged (min–max: 0–4.5). High scores indicate higher levels of physical activity.

#### Body mass Index (BMI)

For part of the sample (*n* = 617) collected in the spring-term 2014, we also asked the participants to self-report their weight (kg) and height (cm). Each participant’s BMI score was calculated using the standard formula weight/height squared (kg/meters^2^).

#### Symptoms of depression (MDI)

Symptoms of depression were measured by applying the Major (ICD-10) Depression Inventory (Bech, Rasmussen, Olsen, Noerholm, & Abildgaard, 2001). This 10-item scale contained one item on sleep problems. This item was removed to avoid conceptual overlap with the insomnia (BIS) scale. The MDI 9-item scale correlated *r* = .99 with the MDI 10-item scale. Response alternatives were 0 (*not at all*), 1 (*some of the time*), 2 (*less than half of the time*), 3 (*more than half of the time*), 4 (*most of the time*), and 5 (*all the time*). Scale scoring closely followed the original instructions (scores were summed). Higher scores indicate more depressive symptomatology (min–max: 0–45). Internal consistency was excellent (*α *= .91).

### Analysis

The analyses were performed in Stata/MP 15.1 for Mac (StataCorp, 2017). We predicted depressive symptoms in two models using Structural Equation Modelling (SEM) analyses with robust standard errors. In the first model, we included gender, insomnia, and physical activity as predictors. We added parental employment status (1 = one or both parents not employed), sexual orientation (1 = sexual minority) as control variables, as prior research has shown that these variables are associated with higher levels of depressive symptoms in this group (Bendixen et al., 2018). In the second model, we did separate predictions for girls and boys. Postestimation procedures for SEM was used for estimating variance (*R*^2^) accounted for by the predictors in the models. Given its statistical and practical advantage compared to the interaction term, the difference in slopes was used to test the moderation effect of gender (Robinson, Tomek, & Schumaker, 2013), and formula (4) in Paternoster et al. (1998) was applied for testing equality of regression (path) coefficients. To test for indirect effects of insomnia and physical activity on depressive symptoms, we performed mediation analyses using the MEDSEM package in Stata (Mehmetoglu, 2017). The significance of indirect effects and the ratio of indirect to total effect (RIT) is reported.

## Results

Sample characteristics and the relationship between the variables separately for boys and girls are shown in Table 1. Girls reported higher level of insomnia compared to boys, *t*(1474) = –6.08, *p *< .001, *d* = –0.32. According to the cut-off criteria described above, 49% of the boys and 62% of the girls had symptom levels indicating possible insomnia. Girls also reported more symptoms of depression than boys, *t*(1424) = –9.14, *p* < .001, *d* = –.49. Boys reported slightly higher levels of physical activity than girls, *t*(1478) = 2.47, *p* =.014, *d* = .13. As shown in Table 1, age was not associated with symptoms of insomnia, physical activity, BMI or to symptoms of depression for either gender. Those who self-reported sexual minority status and having at least one unemployed parent reported more symptoms of depression and insomnia, and lower levels of physical activity. Lower level of exercise was significantly associated with more symptoms of insomnia and depression for girls and boys, and insomnia symptoms showed strong and positive associations with depressive symptoms for girls and boys. These associations were generally stronger for girls than they were for boys. Median BMI was 21.5 and 21.9 for girls and boys respectively, suggesting that this sample of emerging adults aged between 16 and 21 years is largely representative with regard to BMI, as the WHO median BMI for girls and boys aged 17.7 years are 21.2 and 21.5 respectively. Overweight status (BMI > 25) was evident in 12% of the girls and in 17% of the boys. This sample did not differ significantly from the remaining sample (n = 868) and as shown in Table 1, BMI was not significantly associated with any of the relevant variables covered by the study (i.e. depression, physical activity or insomnia) neither among boys nor among girls.

**Table 1.** Sample characteristics and Zero-order (Pearson’s *r*) correlations for girls (*n* = 836) and boys (*n* = 565).

	% Mean (SD)	1	2	3	4	5	6
*Girls*							
(1) Age	17.75 (.98)	–					
(2) Non-heterosexual	12.5%	–.01	–				
(3) Parent(s) unemployed	18.9%	–.01	.04	–			
(4) Insomnia	2.56 (1.48)	.03	.14***	.12**	–		
(5) Physical activity	3.25 (.78)	.04	–.11**	–.15***	–.25***	–	
(6) Depression	16.97 (10.23)	.05	.21***	.14***	.64***	–.26***	–
(7) BMI (*n* = 313)	21.92 (3.16)	.05	.04	−.08	.04	.01	-.06
*Boys*							
(1) Age	17.74 (.93)	–					
(2) Non-heterosexual	8.1%	.02	–				
(3) Parent(s) unemployed	17.7%	.06	.01	–			
(4) Insomnia	2.10 (1.34)	–.06	.06	.10*	–		
(5) Physical activity	3.36 (.78)	.00	–.14***	–.07	–.13**	–	
(6) Depression	12.24 (8.45)	.00	.12**	.11*	.45***	–.14***	–
(7) BMI (*n* = 254)	22.66 (3.99)	.03	-.03	.02	.02	.07	-.01

Note: *p < .05, **p <.01, ***p < .001.

**Figure 1.**
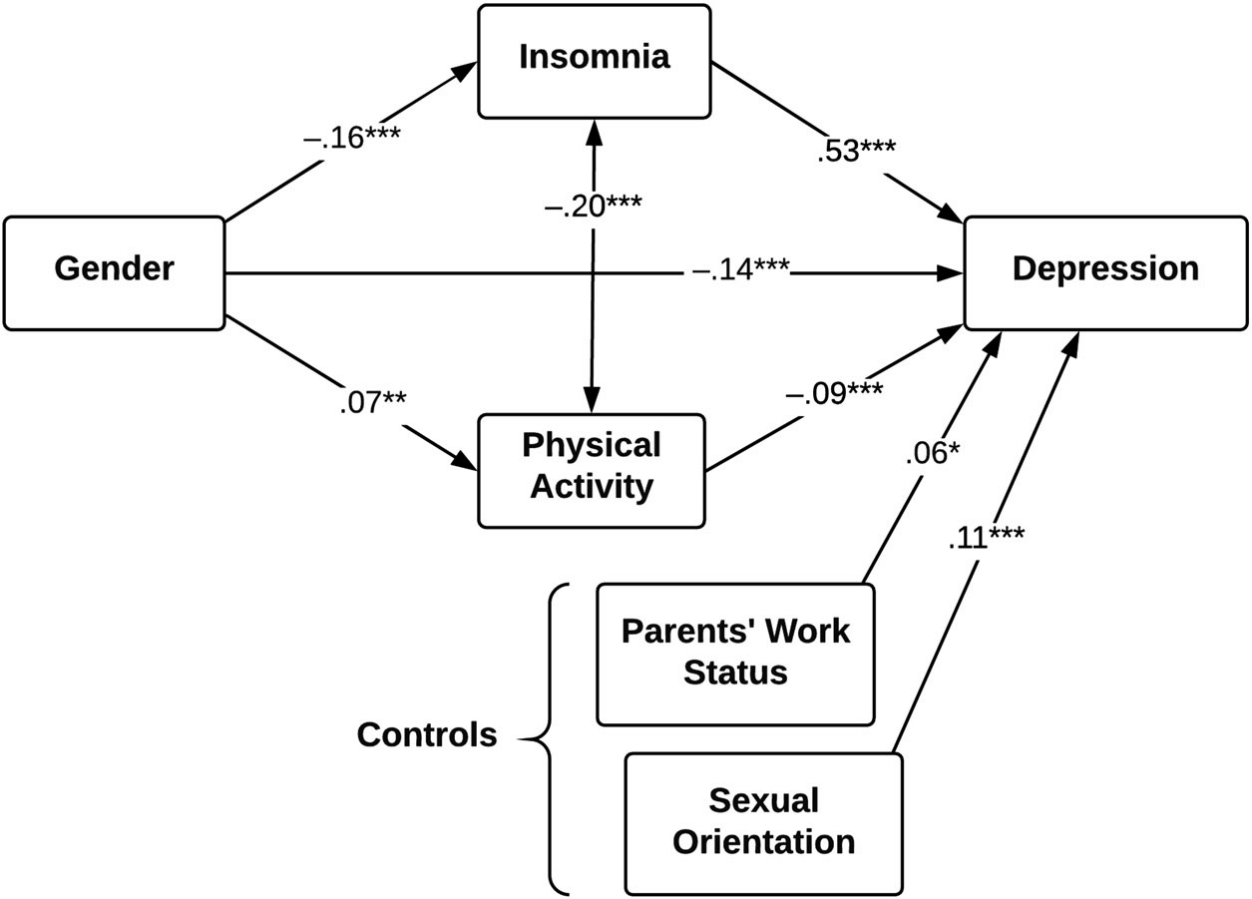
Predictors of symptoms of depression for the full sample (*N* = 1401). * *p* < .05, **p < .01, ***p < .001.

In the structural equation models predicting depressive symptoms, we omitted age and BMI because they showed no association with insomnia, physical activity, or depression. In the first model covering the full sample (Figure 1), being a girl was associated with more symptoms of depression. The gender effect was both a direct (*β* = –.14, *Z* = –6.59, *p* < .001) and indirect (*β* = –.08, *Z* = –5.96, *p* < .001) through its effect on insomnia (*Z* = –6.06, *p* < .001) and to some extent physical activity (*Z* = 2.60, *p* = .009). The principal predictor of depressive symptoms was insomnia. Although the impact of physical activity and the control variables was significant, the effects were generally small. Those reporting lower levels of physical activity also reported more symptoms of insomnia. The variables in the model accounted for 37.4% of the variance (*R*^2^) in depressive symptoms.

**Figure 2.**
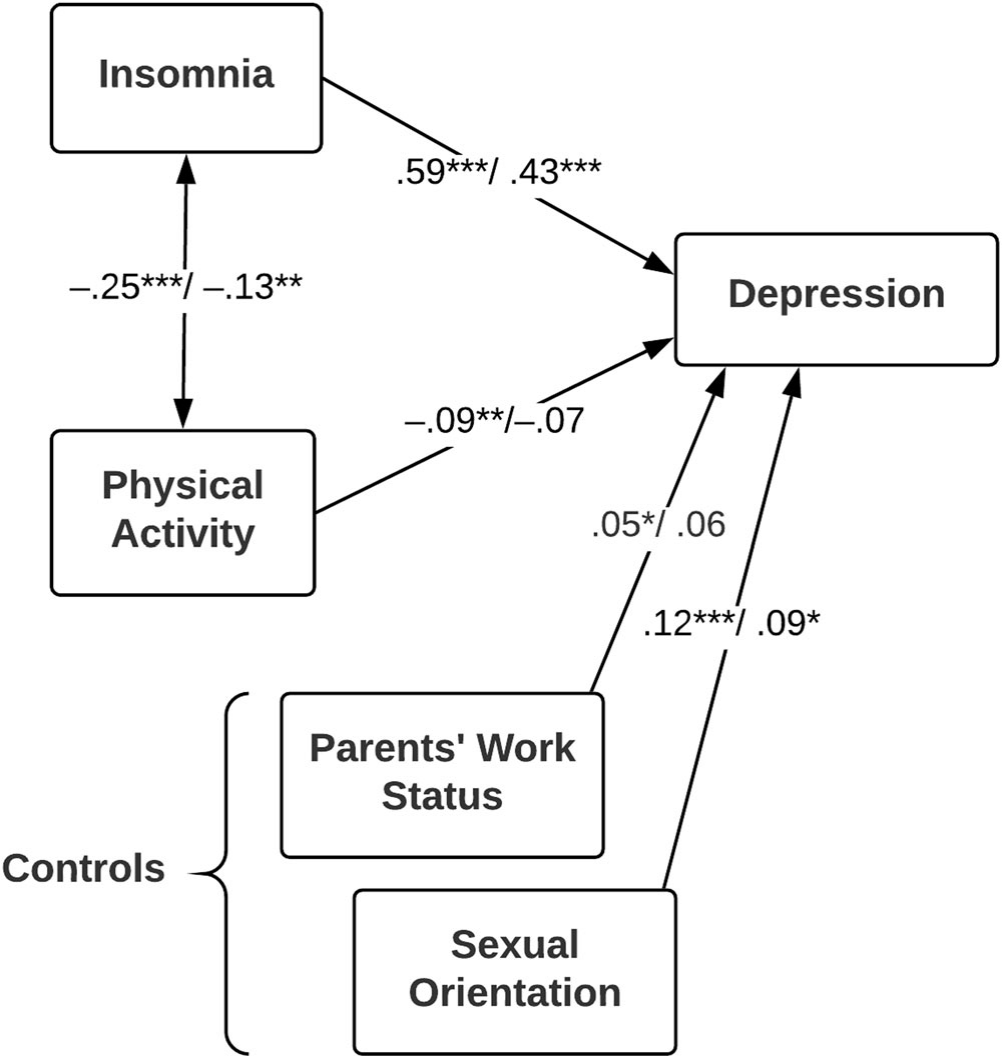
Gender-specific predictors of symptoms of depression. Coefficient are presented as girls (*n* = 836) / boys (*n* = 565). *p < .05, **p < .01, ***p < .001.

In the second model we predicted symptoms of depression separately for girls and boys (Figure 2). Although stronger for girls, insomnia symptoms were the key predictor for depressive symptoms also for boys. The association between insomnia symptoms and physical activity was significantly stronger for girls (*Z* = 2.29, *p* = .022). Lower levels of physical activity significantly predicted more symptoms of depression only for girls. The effect of insomnia on depression was significantly stronger for girls than for boys, *Z* = 4.38, *p* < .001, while the effect of physical activity was not different across gender, *Z* = 0.81, *p* = .42. Hence, gender moderated the relationship between insomnia and symptoms of depression. The variables in the second model accounted for 43.1% of the variance (*R*^2^) in symptoms of depression for girls and 21.7% for boys.

The results from the mediation analysis presented in Table 2 showed that physical activity partly mediated the effect of insomnia on depressive symptoms for girls. However, the mediating effect was marginal and the ratio of indirect to total effect (RIT) was only 4%. On the other hand, insomnia mediated 62% of the effect of physical activity on depressive symptoms for girls. For boys, physical activity did not have a direct effect on depression, only indirectly through its association with insomnia. Physical activity did not mediate the relationship between insomnia and depressive symptoms for boys.

**Table 2.** Direct and indirect effects of the predictors on symptoms of depression in the structural model (girls, *n* = 836 and boys, *n* = 565).

		Direct effects			Indirect effects through insomnia				Indirect effects through physical activity			
	Predictor	*β*	*p*	95% CI	*β*	*p*	95% CI	RIT	*β*	*p*	95% CI	RIT
Girls												
	Insomnia	.59	<.001	[ .55, .64]					.02	.004	[ .01, .04]	.04
	Physical activity	–.09	.002	[–.15, .03]	–.15	<.001	[–.19, –.11]	.62				
	Parents’ work status	.05	.044	[ .00, .10]								
	Sexual orientation	.12	<.001	[ .06, .17]								
Boys												
	Insomnia	.43	<.001	[ .36, .50]					.01	.16	[–.00, .02]	.02
	Physical activity	–.07	.093	[–.15, .01]	–.06	.004	[–.09, –.02]	.45				
	Parents’s work status	.06	.157	[–.02, .15]								
	Sexual orientation	.09	.049	[ .00, .17]								

Note: RIT = Ratio of indirect to total effect.

## Discussion

In this large, community sample of high-school students, self-reported insomnia was highly prevalent, especially among girls. Age was not significantly associated with depressive symptoms in this restricted age range. Further, there was a strong association between symptoms of insomnia and symptoms of depression, and this association was especially strong for girls. Physical activity was significantly associated with depression for both boys and girls. This finding is in accordance with previous research (Camero et al., 2012; Jerstad et al., 2010; Kremer et al., 2014). In the SEM analysis, this association remained significant only for girls after controlling for insomnia. Also, physical activity moderated the effect of insomnia only for girls, and this effect was marginal.

### The importance of a gender-specific approach

Consistent with prior findings (e.g. Baldursdottir et al., 2017), girls reported a significantly lower level of physical activity compared to boys. Further, girls reported substantially higher levels of depression and insomnia, and the relationships between physical activity and these two variables were stronger for girls compared to boys. Although the negative association between physical activity and insomnia observed in this study is consistent with prior studies (e.g. Kredlow et al., 2015; Youngstedt & Kline, 2006), our findings suggest that this association was stronger for girls than for boys. Although familial history of mental disorder, early childhood trauma, and functional impairment have been identified as important predictors of depression among both boys and girls (Whalen et al., 2016), the increasing gender-differences during the period of adolescence (Piccinelli & Wilkinson, 2000; Sequeira et al., 2017) suggest that some predictors of depression might have a gender-specific effect.

In our present study, insomnia was a significantly stronger predictor of symptoms of depression among girls compared to boys. Compared to physical activity, insomnia was a stronger predictor of depression for both boys and girls. Interestingly, overweight was not associated with symptoms of depression, contrary to other studies finding that obesity was a general (Cho et al., 2018) and gender-specific predictor of depression (Mulugeta et al., 2018). Screening for sleep problems has been suggested as an opportunity for detecting and reducing the burden of mental illness in children and adolescents (Shanahan et al., 2014). As insomnia represents a major challenge to mental health, even as an independent entity (de Zambotti et al., 2018), interventions aimed at reducing sleep problems among adolescents are possible avenues to reducing depressive symptoms in general, but maybe especially so for adolescent girls and emerging adult women.

### Implications

The results strongly suggest that addressing sleep-disorders among adolescents should be incorporated in health-promotion interventions aimed at reducing symptoms of depression. Research has shown that internet-delivered interventions aimed at reducing insomnia are effective (Hagatun et al., 2018), and hence offering a promising avenue for addressing insomnia as a health challenge among adolescents. The effect of insomnia on depression was both direct and indirect for both boys and girls, while the effect of physical activity was marginal, especially for boys, where physical activity only had an indirect effect through insomnia. This study highlights the importance of incorporating sleep into research on the effect of physical activity on depressive symptoms. However, among girls, the SEM-analysis showed that physical activity had a direct, and indirect, although marginal, the effect on symptoms of depression. This suggests that interventions aimed at reducing sedentary behavior, especially among girls, also could benefit on their own terms. The reason for the stronger association between insomnia and depression observed for girls should be further investigated in longitudinal studies exploring gender-specific determinants of insomnia.

### Strengths and limitations

The cross-sectional nature of this study warrants caution when it comes to inferring causality. In survey-designs, common-method bias will represent a possible threat to the validity. Further, the operational definition of physical activity also limits firm conclusions, as it relies on self-report of participation in mandatory and elective physical activities. This measure does not account for the intensity or duration of the physical activity. Hence, future studies should preferably make use of additional objective measures of both sleep and activity, like actigraphy, in a longitudinal design, preferably with the combination of measures of sleep-quality (e.g. sleep-diaries). The measures of insomnia and depression are validated and is a strength in this study of a large, representative sample.

### Conclusion

Given the substantial gender-differences in depression, insomnia and physical activity, there is a need to regard adolescent girls and boys as different populations when investigating the association between these variables. In this large-scale study of a representative community sample, the association between insomnia and symptoms of depression was significantly stronger for girls compared to boys. Physical activity was independently associated with symptoms of depression when controlling for insomnia, but only for girls, and of substantially smaller magnitude. Further, insomnia mediated the association between physical activity. Understanding gender-differences in the risk of developing insomnia could inform research on gender differences in depression. There is a need for longitudinal designs investigating the gender-specific reciprocal association between insomnia, physical activity and symptoms of depression among adolescents. The results suggest that interventions aimed at targeting insomnia among adolescents could serve as a promising avenue for promoting mental health.
